# “MY LIFE WAS EASY; HIS LIFE TAKES ALL OF MY TIME”: THE INTERPLAY OF SUPPORT STRUCTURES AND DEMENTIA CARE NEEDS IN THE HOME

**DOI:** 10.1093/geroni/igae098.2178

**Published:** 2024-12-31

**Authors:** Theresa Allison, Alexander Smith, Jennie Gubner, Kenneth Covinsky

**Affiliations:** University of California San Francisco, San Francisco, California, United States; University of California San Francisco, San Francisco, California, United States; University of Arizona, Tucson, Arizona, United States; University of California San Francisco, San Francisco, California, United States

## Abstract

We have limited literature about the lived realities of in-home dementia care. Using data from two ethnographic studies of music and everyday life in the homes of people living with dementia, we investigated the relationship between care support resources and multimorbidity care needs. Data included demographics, measures, fieldnotes and semi-structured interview transcripts. We inductively coded transcripts using ATLAS.ti and triangulated findings across data sources. N=83 (38 people living with dementia, 34 family care partners, 11 professionals). Participation varied from a single phone interview to 8 in-home visits over two years. Participants identified complex mismatches between resources and care needs, particularly in ambulatory stages of dementia or with care partner multimorbidity. In contrast, participants identified spirituality as a powerful motivator for providing care. Participants identified adequate social support and financial resources as critical. The findings underscore the need for nuanced approaches to interventions, acknowledging both positive and negative aspects of caregiving.

